# Renal Function Declines More in Tenofovir- than Abacavir-Based Antiretroviral Therapy in Low-Body Weight Treatment-Naïve Patients with HIV Infection

**DOI:** 10.1371/journal.pone.0029977

**Published:** 2012-01-05

**Authors:** Takeshi Nishijima, Hiroyuki Gatanaga, Hirokazu Komatsu, Kunihisa Tsukada, Takuro Shimbo, Takahiro Aoki, Koji Watanabe, Ei Kinai, Haruhito Honda, Junko Tanuma, Hirohisa Yazaki, Miwako Honda, Katsuji Teruya, Yoshimi Kikuchi, Shinichi Oka

**Affiliations:** 1 AIDS Clinical Center, National Center for Global Health and Medicine, Tokyo, Japan; 2 Department of Clinical Research and Informatics, International Clinical Research Center, National Center for Global Health and Medicine, Tokyo, Japan; 3 Department of Community Care, Saku Central Hospital, Nagano, Japan; 4 Center for AIDS Research, Kumamoto University, Kumamoto, Japan; UCL Institute of Child Health, University College London, United States of America

## Abstract

**Objective:**

To compare the rate of decline of renal function in tenofovir- and abacavir-based antiretroviral therapy (ART) in low-body weight treatment-naïve patients with HIV infection.

**Design:**

We conducted a single-center retrospective cohort study of 503 Japanese patients who commenced on either tenofovir- or abacavir-based initial ART.

**Methods:**

The incidence of renal dysfunction, defined as more than 25% fall in estimated glomerular filtration rate (eGFR) from the baseline, was determined in each group. The effect of tenofovir on renal dysfunction was estimated by univariate and multivariate Cox hazards models as the primary exposure. Changes in eGFR until 96 weeks were estimated in both groups with a repeated measures mixed model.

**Results:**

The median body weight of the cohort was 64 kg. The estimated incidence of renal dysfunction in the tenofovir and the abacavir arm was 9.84 per 100 and 4.55 per 100 person-years, respectively. Tenofovir was significantly associated with renal dysfunction by univariate and multivariate analysis (HR = 1.747; 95% CI, 1.152–2.648; p = 0.009) (adjusted HR = 2.080; 95% CI, 1.339–3.232; p<0.001). In subgroup analysis of the patients stratified by intertertile baseline body weight, the effect of tenofovir on renal dysfunction was more evident in patients with lower baseline body weight by multivariate analysis (≤60 kg: adjusted HR = 2.771; 95%CI, 1.494–5.139; p = 0.001) (61–68 kg: adjusted HR = 1.908; 95%CI, 0.764–4.768; p = 0.167) (>68 kg: adjusted HR = 0.997; 95%CI, 0.318–3.121; p = 0.995). The fall in eGFR was significantly greater in the tenofovir arm than the abacavir arm after starting ART (p = 0.003).

**Conclusion:**

The incidence of renal dysfunction in low body weight patients treated with tenofovir was twice as high as those treated with abacavir. Close monitoring of renal function is recommended for patients with small body weight especially those with baseline body weight <60 kg treated with tenofovir.

## Introduction

Tenofovir disoproxil fumarate (TDF) and abacavir sulfate (ABC) are widely used nucleot(s)ide reverse transcriptase inhibitors (NRTIs) as part of the initial antiretroviral therapy for patients with HIV infection in the developed countries (URL:http://www.aidsinfo.nih.gov/ContentFiles/AdultandAdolescentGL.pdf) (URL:http://www.europeanaidsclinicalsociety.org/images/stories/EACS-Pdf/1_treatment_of_hiv_infected_adults.pdf). TDF is generally preferred to ABC, since ABC is reported to cause serious hypersensitivity reaction in 5–8% of the patients and its efficacy in viral suppression is reported to be inferior to TDF among patients with baseline HIV viral load of >100,000 copies/ml [1], [2]. On the other hand, renal proximal tubular damage and renal dysfunction are well-known adverse effects of TDF [3]–[9]. A meta-analysis study that compared TDF and other NRTIs concluded that the decline in renal function with TDF use is significant but modest, and the ASSERT study conducted in Europe compared randomly-selected treatment naïve patients who commenced treatment with either TDF or ABC with efavirenz and showed no difference in estimated glomerular filtration rate (eGFR) between the two groups at 48 weeks [9], [10]. To date, the nephrotoxicity of TDF have been regarded as mild and tolerable [2], [5]–[7], [9]–[11].

However, the TDF-related nephrotoxicity has hardly been evaluated in patients with small body weight, who are potentially at higher risk for larger drug exposure and thus, more severe toxicity [12]–[15]. Indeed, some recent studies including ours reported a higher incidence of TDF-related renal dysfunction among Asian patients with low body weight compared with previous studies on mostly Whites and African Americans with larger body weight [13], [16]. Thus, it is important to provide more evidence in support of TDF-associated nephrotoxicity in patients with low body weight since such data can elucidate whether TDF-related nephrotoxicity is as mild in low-body-weighted patients as previously reported in Europe and the USA. This is also important because there is increasing use of TDF in resource-limited settings, where patients are often of relatively small body weight, following the revised 2010 WHO guidelines that recommend TDF as one of the components of first line therapies (URL:http://whqlibdoc.who.int/publications/2010/9789241599764_eng.pdf)[13], [16]–[19]. To our knowledge, there are no studies that compared renal function in treatment naïve Asian patients who commenced treatment with TDF or ABC.

Based on the above background, the present study was designed to compare the incidence of renal dysfunction and change in eGFR between treatment-naïve Japanese patients with low body weight who started either TDF or ABC as part of the antiretroviral regimen.

## Methods

### Ethics Statement

This study was approved by the Human Research Ethics Committee of National Center for Global Health and Medicine, Tokyo. All patients included in this study provided a written informed consent for their clinical and laboratory data to be used and published for research purposes. This study has been conducted according to the principles expressed in the Declaration of Helsinki.

### Study Subjects

We performed a retrospective, single-center cohort study of HIV-infected Japanese patients using the medical records at the National Center for Global Health and Medicine, Tokyo, Japan. Our facility is one of the largest clinics for patients with HIV infection in Japan with more than 2,700 registered patients. The study population was treatment-naïve patients with HIV infection, aged >17 years, who commenced treatment with either the recommended 300 mg/day dose of TDF or 600 mg/day dose of ABC-containing antiretroviral regimen at our clinic between January 1, 2004 and March 31, 2009. During this inclusion period, all except two patients at our clinic started ART with either ABC or TDF. Patients with an eGFR of >60 ml/min/1.73 m^2^ were enrolled. Patients were followed up until March 31, 2011. They were excluded if they started ART with both TDF and ABC, their follow-up period at our facility was less than 24 weeks after commencement of ART, or if they had started ART at other facilities. Only Japanese patients were included in order to examine a population with comparatively homogenous basic demographics and background. The attending physician selected either TDF or ABC at baseline, and the use of these two drugs was based on the Japanese guidelines, which place both ABC and TDF as the preferred NRTIs (http://www.haart-support.jp/guideline2011.pdf. in Japanese). The attending physician also selected the key drug [non-nucleoside reverse transcriptase inhibitor (NNRTI), protease inhibitor (PI), or integrase inhibitor (INI)]. All patients received standard ART with two NRTIs combined with either PI, NNRTI, or INI.

### Measurements

We defined renal dysfunction as more than 25% decrease in eGFR relative to the baseline [13], [16], [20], [21]. The baseline eGFR was estimated for each patient from the average of two successive serum creatinine measurements made closest to and preceding the commencement of antiretroviral therapy by no more than 90 days. Changes in eGFR were plotted from the baseline measurement until the average value of two successive measurements diminished to less than 75% of the baseline, discontinuation of TDF or ABC, or at the end of the follow-up period. Discontinuation of TDF and ABC was the choice of the attending physician, and was based on virologic failure or ART-related side effects other than renal dysfunction. Before the initiation of ART and until suppression of HIV-1 viral load, patients visited our clinic every month. However, after viral load suppression, the visit interval was extended up to every three months. Serum creatinine and eGFR were measured in every visit, and the frequency of measurements was similar in patients on TDF and ABC. eGFR was calculated using the equation from the 4-variable Modification of Diet in Renal Disease (MDRD) study, eGFR = 186× [serum creatinine]^−1.154^× [age]^−0.203^× [0.742 if patient is female] × [1.212 if patient is African American] [22]. In this study, the primary exposure variable was TDF use over ABC as part of the initial ART.

The potential risk factors for renal dysfunction were determined according to previous studies and collected together with the basic demographics from the medical records [15], [23]–[25]. They included age, sex, body weight, body mass index, (BMI)  =  {body weight (kg) / [(height (m)]^2^}, baseline laboratory data (CD4 cell count, HIV viral load, and serum creatinine), and presence or absence of other medical conditions (concurrent use of ritonavir-boosted protease inhibitors, concurrent nephrotoxic drugs such as ganciclovir, sulfamethoxazole/trimethoprim, and non-steroidal anti-inflammatory agents, diabetes mellitus defined by using anti-diabetic agents or fasting plasma glucose >126 mg/dl or plasma glucose >200 mg/dl on two different days, co-infection with hepatitis B defined by positive hepatitis B surface antigen, co-infection with hepatitis C defined by positive HCV viral load, hypertension defined by current treatment with antihypertensive agents or two successive measurements of systolic blood pressure >140 mmHg or diastolic blood pressure >90 mmHg at the clinic, dyslipidemia defined by current treatment with lipid-lowering agents, and current smoking). At our clinic, weight and blood pressure were measured on every visit whereas other variables were measured in the first visit and at least once annually. We used the data on or closest to and preceding the day of starting ART by no more than 90 days.

### Statistical analysis

The time to 25% decline in eGFR from the baseline was calculated from the date of commencement of treatment to the date of diagnosis of the above-defined renal dysfunction. Censored cases represented those who discontinued ABC or TDF, dropped out, were referred to other facilities, or at the end of follow-up period. The time from the start of ART to >25% decrease in eGFR was analyzed by the Kaplan Meier method for patients who started TDF (TDF arm) and ABC (ABC arm), and the log-rank test was used to determine the statistical significance. The Cox proportional hazards regression analysis was used to estimate the impact of TDF use over ABC on the incidence of more than 25% decrease in eGFR relative to the baseline. The impact of each basic demographics, baseline laboratory data, and other medical conditions listed above was also estimated with univariate Cox proportional hazards regression.

To estimate the unbiased prognostic impact of TDF use over ABC for renal dysfunction, we conducted three models using multivariate Cox proportional hazards regression analysis. Model 1 was the aforementioned univariate analysis for TDF use over ABC. Model 2 included age and weight plus model 1 in order to adjust for basic characteristics. In model 3, we added variables with P values <0.05 in univariate analysis for adjustment (these included age per 1 year, weight per 1 kg decrement, CD4 count per 1 /µl decrement, HIV viral load per log10/ml, serum creatinine per 1 mg/dl, concurrent use of nephrotoxic drug(s), hepatitis B infection, and diabetes mellitus). The eGFR and the BMI were excluded from multivariate analysis because of their multicollinearity with age and serum creatinine, and weight, respectively, since eGFR and BMI are gained by the equation of those variables [22], [26]. We chose to add weight instead of BMI because our previous work showed that weight was more useful and handy information to estimate the risk for TDF-related nephrotoxicity than BMI [16].

As a sensitivity analysis, creatinine clearance was similarly calculated with Cockcroft-Gault equation for each patient, creatinine clearance  =  [(140 - age) × weight (kg)] / (serum creatinine×72)(×0.85 for females) [27]. Actual body weight was used for the calculation. The impact of TDF use over ABC for >25% decrement of creatinine clearance from the baseline was estimated in univariate analysis and multivariate analysis adjusted with the before mentioned variables with Cox proportional hazards model.

To estimate the impact of weight on TDF-related nephrotoxicity, we did subgroup analysis for intertertile baseline body weight categories: ≤60, 61–68, and >68 kg. Then, the abovementioned multivariate analysis with eGFR was conducted for each subgroup.

We also used a repeated measures mixed model to estimate and compare changes in eGFR between ABC and TDF from baseline to 2 years after initiation of ART by 6-month intervals adjusted for baseline eGFR and weight [10]. For each patient, the eGFR values at closest to and preceding 24, 48, 72 and 96 weeks after commencement of ART were collected. In this analysis, censoring occurred at discontinuation of TDF or ABC, leaving care, or reaching the end of the observation period before 96 weeks. Sensitivity analysis with creatinine clearance calculated by Cockcroft-Gault equation was similarly conducted.

Statistical significance was defined at two-sided *p* values <0.05. We used hazard ratios (HRs) and 95% confidence intervals (95% CIs) to estimate the impact of each variable on renal dysfunction. All statistical analyses were performed with The Statistical Package for Social Sciences ver. 17.0 (SPSS, Chicago, IL).

## Results

The study subjects were 199 patients in the TDF arm and 304 patients in the ABC arm who fulfilled the abovementioned criteria. Table 1 shows the demographics, laboratory data, and medical conditions of the study population at baseline. The majority of the study population was males, comparatively young and had a small stature (median weight, 64 kg, median BMI, 22.2 kg/m^2^). More than 80% of the patients in the two arms had ritonavir-boosted PI. In the ABC arm, patients had significantly lower CD4 count (p = 0.006), were significantly more likely to have hypertension (p<0.001), and tended to use more nephrotoxic drugs (p = 0.109). On the other hand, in the TDF arm, patients had marginally higher baseline eGFR (p = 0.098) and were significantly more likely to have hepatitis B virus infection (P<0.001). However, all other major background parameters were similar in the two groups (Table 1).

**Table 1:** Baseline demographics and laboratory data of patients who received tenofovir- and abacavir-based antiretroviral therapy (n = 503).

	TDF (n = 199)	ABC (n = 304)	P value
Sex (male), n (%)	196 (98.5)	296 (97.4)	0.539
Median (IQR) age	36 (31–44)	37 (31–43)	0.436
Median (IQR) weight (kg)	64 (58–69)	64 (58.0–70.9)	0.426
Median (IQR) BMI (kg/m^2^)	22.1 (20.4–23.9)	22.2 (20.3–24.6)	0.321
Median (IQR) eGFR (ml/min/1.73m^2^)	119.4 (103.0–135.0)	115.6 (102.4–132.2)	0.098
Median (IQR) serum creatinine (mg/dl)	0.74 (0.67–0.84)	0.75 (0.68–0.83)	0.250
Median (IQR) CD4 count (/µl)	199 (109–272)	178.5 (75.3–234.8)	0.006
Median (IQR) HIV RNA viral load (log10/ml)	4.63 (4.20–5.20)	4.74 (4.23–5.20)	0.731
Ritonavir-boosted protease inhibitors, n (%)	173 (86.9)	256 (84.2)	0.441
Protease inhibitors (unboosted), n (%)	5 (2.5)	20 (6.6)	0.038
NNRTIs, n (%)	16 (8.0)	26 (8.6)	0.848
INIs, n (%)	5 (2.5)	2 (0.7)	0.119
Hypertension, n (%)	5 (2.5)	53 (17.4)	<0.001
Dyslipidemia, n (%)	4 (2.0)	4 (1.3)	0.718
Diabetes mellitus, n (%)	8 (4.0)	12 (3.9)	1.000
Concurrent use of nephrotoxic drugs, n (%)	65 (32.7)	121 (39.8)	0.109
Hepatitis B, n (%)	35 (17.6)	9 (3.0)	<0.001
Hepatitis C, n (%)	7 (3.5)	7 (2.3)	0.421
Current smoker, n (%)	93 (46.7)	149 (49.3)	0.585

TDF: tenofovir, ABC: abacavir, IQR: interquartile range, BMI: body mass index, eGFR: estimated glomerular filtration rate, NNRTI: non- nucleoside reverse transcriptase inhibitor, INI: integrase inhibitor.

**Figure 1:**
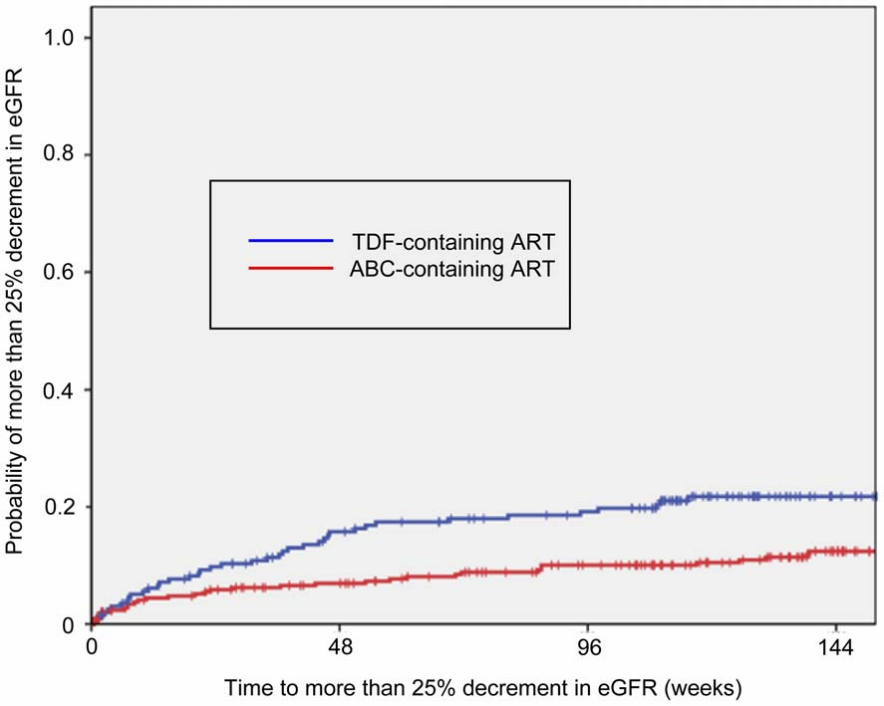
Kaplan-Meier curve showing the time to renal dysfunction in patients treated with TDF or ABC. Compared to treatment-naïve patients who commenced treatment with ABC, those on TDF were more likely to develop >25% fall in eGFR (p = 0.001, Log-rank test). TDF: tenofovir, ABC: abacavir, ART: antiretroviral therapy, eGFR: estimated glomerular filtration rate.

More than 25% decrement in eGFR from baseline occurred in 44 patients (22.1%) in the TDF arm and 41 (13.5%) in the ABC arm, with an estimated incidence of 9.84 and 4.55 per 100 person-years, respectively. Figure 1 shows the time from ART initiation to >25% decrease in eGFR by the Kaplan Meier method in the two groups. Patients who started TDF-containing ART were significantly more likely to develop renal dysfunction, compared to the ABC group (p = 0.001, Log-rank test). The median time from commencement of ART to occurrence of >25% decrement in eGFR was 246 days (range, 1–1,339 days) for the TDF arm and 501 days (range, 7–2,022) for ABC arm. The total observation period was 447.2 patient-years [median, 839 days, interquartile range (IQR), 357–1137 days] for the TDF arm and 901.7 patient-years (median, 1,119 days, IQR, 660.5–1509 days) for the ABC arm.

Univariate analysis showed a significant relationship between TDF use and >25% decrement in eGFR (HR = 1.747; 95%CI, 1.152–2.648; p = 0.009) (Table 2). Furthermore, old age, small body weight, low baseline CD4 count, high HIV viral load, high eGFR, low serum creatinine, concurrent use of nephrotoxic drugs, hepatitis B infection, and diabetes mellitus were associated with renal dysfunction. On the other hand, concurrent use of ritonavir boosted PIs was not associated with renal dysfunction (HR = 1.220; 95%CI, 0.663–2.244; p = 0.523). Multivariate analysis identified TDF use as a significant risk for >25% decrement in eGFR after adjustment for age and weight (adjusted HR = 1.893; 95%CI, 1.243–2.881; p<0.003) (Table 3, Model 2), and also after adjustment for other risk factors (adjusted HR = 2.080; 95%CI, 1.339–3.232; p<0.001) (Table 3, Model 3). We also conducted a sensitivity analysis using BMI decrement instead of weight as a variable in Table 3, Model 3. The results were almost identical; TDF use over ABC use was a risk for renal dysfunction (adjusted HR 1.957, 95% CI 1.262–3.036, p = 0.003).

**Table 2:** Univariate analysis to estimate the risk of various factors in inducing more than 25% fall in eGFR.

	Hazard ratio	95% CI	P value
TDF vs. ABC use	1.747	1.152–2.648	0.009
Female gender	0.048	0.000–16.93	0.310
Age per 1 year	1.031	1.011–1.051	0.002
Weight per 1 kg decrement	1.047	1.023–1.072	<0.001
BMI per 1 kg/m^2^ decrement	1.152	1.066–1.244	<0.001
CD4 count per 1 /µl decrement	1.006	1.004–1.008	<0.001
HIV viral load per log10/ml	1.562	1.179–2.071	0.002
Ritonavir-boosted protease inhibitors	1.220	0.663–2.244	0.523
Baseline eGFR per 1 ml/min/1.73m^2^	1.009	1.005–1.014	<0.001
Baseline serum creatinine per 1mg/dl	0.016	0.003–0.086	<0.001
Concurrent nephrotoxic drug	2.134	1.417–3.214	<0.001
Hepatitis B	1.866	1.038–3.356	0.037
Hepatitis C	1.721	0.631–4.695	0.289
Diabetes mellitus	2.558	1.181–5.540	0.017
Hypertension	0.865	0.448–1.669	0.664
Current smoking	0.989	0.657–1.489	0.958

eGFR: estimated glomerular filtration rate, CI: confidence interval, TDF: tenofovir, ABC: abacavir, BMI: body mass index.

**Table 3:** Multivariate analysis to estimate the risk of TDF- over ABC-based antiretroviral therapy in inducing more than 25% fall in eGFR.

	Model 1 Crude		Model 2 Adjusted		Model 3 Adjusted	
	HR	95% CI	HR	95%CI	HR	95%CI
TDF vs. ABC use†	1.747	1.152–2.648	1.893	1.243–2.881	2.080	1.339–3.232
Age per 1 year			1.029	1.010–1.048	1.020	1.000–1.040
Weight per 1 kg decrement†			1.046	1.022–1.071	1.028	1.005–1.052
CD4 count per 1 /µl decrement†					1.004	1.002–1.007
HIV viral load per log10/ml					1.048	0.749–1.466
Serum creatinine per 1 mg/dl†					0.053	0.009–0.304
Use of nephrotoxic drug					1.309	0.825–2.077
Hepatitis B					1.070	0.573–2.000
Diabetes mellitus					1.565	0.684–3.582

† P<0.05 in Model 3.

TDF: tenofovir, ABC: abacavir, eGFR: estimated glomerular filtration rate, HR: hazard ratio, CI: confidence interval.

Sensitivity analysis with creatinine clearance confirmed the abovementioned findings: both univariate and multivariate analyses showed that TDF use was significantly associated with >25% decrement in eGFR (univariate analysis: HR = 2.212; 95%CI, 1.340–3.653; p = 0.002) (multivariate analysis: adjusted HR = 2.544; 95%CI, 1.493–4.335; p = 0.001).

Subgroup analysis of the patients stratified by intertertile baseline body weight showed that the lower the baseline body weight, the more evident the impact of TDF on renal dysfunction (≤60 kg: adjusted HR = 2.771; 95%CI, 1.494–5.139; p = 0.001) (61–68 kg: adjusted HR = 1.908; 95%CI, 0.764–4.768; p = 0.167) (>68 kg: adjusted HR = 0.997; 95%CI, 0.318–3.121; p = 0.995) (Table 4). These findings suggest that there is the effect modification by baseline body weight on TDF-associated renal dysfunction.

**Table 4:** Multivariate analysis to estimate the risk of TDF- over ABC-based antiretroviral therapy in the induction of more than 25% fall in eGFR according to baseline body weight.

	Adjusted HR	95% CI	P value
Baseline body weight ≤60 kg (n = 171)			
TDF vs. ABC use	2.771	1.494–5.139	0.001
Baseline body weight 61–68 kg (n = 167)			
TDF vs. ABC use	1.908	0.764–4.768	0.168
Baseline body weight >68 kg (n = 165)			
TDF vs. ABC use	0.997	0.318–3.121	0.995

TDF use was adjusted with the same variables indicated in Model 3, Table 3: age per 1 year, weight per 1 kg decrement, CD4 count per 1 /µl decrement, HIV viral load per log10/ml, serum creatinine per 1 mg/dl, concurrent use of nephrotoxic drugs, hepatitis B infection, and diabetes mellitus.

**Figure 2:**
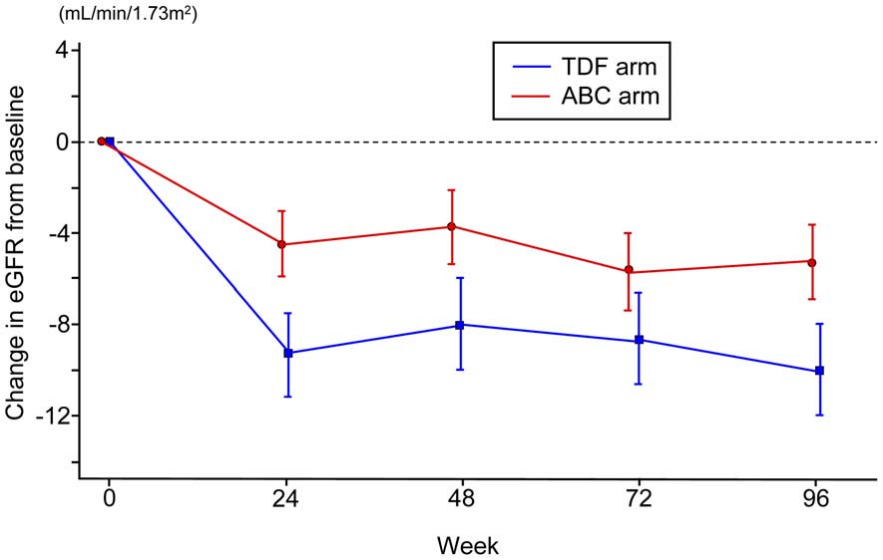
Changes in eGFR in patients treated with TDF or ABC between baseline and 96 weeks. The fall in eGFR was significantly greater in the TDF group than the ABC group (p = 0.003). Data are adjusted mean ±95% confidence interval. eGFR: estimated glomerular filtration rate, TDF: tenofovir, ABC: abacavir.

Data analysis by repeated measures mixed models showed a significant decrease in adjusted mean eGFR from the baseline to 96 weeks in both groups (TDF: -9.984 ml/min/1.73m^2^, 95%CI -12.05 to -7.914 ml/min/1.73m^2^, p<0.001; ABC: -5.393 ml/min/1.73m^2^, 95%CI -7.087 to -3.699 ml/min/1.73m^2^, p<0.001) (Figure 2). There was a statistically significant interaction between the two arms over time (p = 0.003), indicating that adjusted mean eGFR decreased more significantly in the TDF group than in the ABC group after initiation of ART. Analysis of eGFR in each group demonstrated a rapid decrease during the first 24 weeks, followed by a plateau until 96 weeks. In sensitivity analysis with creatinine clearance calculated by Cockcroft-Gault equation, the result was the same; a significant decrease from the baseline to 96 weeks in both groups (TDF: -10.62 ml/min, 95%CI -13.78 to -7.458 ml/min; ABC: -4.325 ml/min, 95%CI -6.893 to -1.756 ml/min) and significantly more eGFR decrement in the TDF group (p = 0.019).

## Discussion

In this observational Japanese cohort, treatment-naïve patients who started TDF-containing ART experienced eGFR decline of >25% approximately twice as likely compared to those treated with ABC-containing regimen. Univariate and multivariate analyses identified TDF use as an independent risk factor for renal dysfunction. Subgroup analysis showed that the effect of TDF on renal dysfunction was more evident in patients with lower body weight. Furthermore, eGFR decrement was significantly larger in the TDF group than in ABC group over the 2-year observation period.

In our previous study, we demonstrated a high incidence of TDF-associated nephrotoxicity in patients with low body weight, and the use of a robust statistical model indicated a greater decline in renal function in patients of low body weight treated with TDF [16]. The results of the present study further emphasize the importance of low body weight as a risk factor for TDF-related nephrotoxicity by showing that in a cohort of patients with low body weight, the incidence of renal dysfunction was twice higher with TDF use than with ABC use.

Among the studies designed to compare renal function after the commencement of TDF and ABC-containing ART for treatment-naïve patients, our cohort had the lowest median body weight (64 kg). This is lower than the median body weight of patients of the ASSERT study conducted in European countries (72 kg) [10]. The results of the present study on TDF-related nephrotoxicity differ from the findings of randomized clinical trials that demonstrated no major change in renal function of TDF- and ABC-treated patients over 48–96 week follow-up [2], [10], [11]. The discrepant results might arise from differences between observational cohort and clinical trials, since observational studies tend to express the results in “real world setting” whereas clinical trials include patients who fulfill more strict criteria, therefore with better profile [9]. The discrepant results could be also due to the use of different definitions for renal dysfunction in these studies. However, the discrepant results could also reflect the difference in median body weight between the present study and these clinical trials. The results of our subgroup analysis support this hypothesis by showing that the effect of TDF on renal dysfunction was more evident in patients with low body weight. Apart from being low-body-weighted, the patients in this study did not appear to have many of other established risks for TDF-related nephrotoxicity; they were comparatively young, had relatively stable CD4 count, and had only a few co-morbidities (Table 1). Although the majority concurrently used ritonavir-boosted PIs, which are a probable risk for TDF-related nephrotoxicity, ritonavir-boosted PIs were not significantly associated with renal dysfunction in our cohort (Table 2) [24].

Changes in eGFR in those patients treated with TDF-containing ART were characterized by a rapid decline during the first 24 weeks of therapy, followed by a plateau until 96 weeks (Fig. 2). This finding is consistent with that reported from the Johns Hopkins group [9], [28]. Together with the finding that the median time from commencement of ART to the >25% decline in eGFR in the TDF-treated patients was 246 days, these results suggest that careful monitoring of renal function is particularly warranted in the first year of TDF-based therapy. Thus, we suggest that renal function should be monitored by measurement of serum creatinine at least once annually in resource-limited settings and twice annually in resource-rich settings in patients starting TDF-containing ART, especially those with baseline body weight <60 kg.

The Department of Health and Human Services guideline for the treatment of HIV infection in the U.S. lists ABC as an alternative NRTI because it can potentially cause serious hypersensitivity reaction and cardiovascular diseases (URL:http://www.aidsinfo.nih.gov/ContentFiles/AdultandAdolescentGL.pdf). However, some international guidelines consider both TDF and ABC as the preferred NRTIs under the condition that ABC should be used with caution in patients with viral load >100,000 copies/mL, based on the low incidence of ABC-related hypersensitivity among HLA-B*5701-negative population and the controversial association between ABC and cardiovascular diseases [1], [29]–[32] (URL:http://www.europeanaidsclinicalsociety.org/images/stories/EACS-Pdf/1_treatment_of_hiv_infected_adults.pdf) (http://www.haart-support.jp/guideline2011.pdf. in Japanese). The present study, together with our previous analysis that demonstrated preferential TDF-related nephrotoxicity in patients with low body weight, emphasize the advantage of ABC over TDF with regard to prognosis of renal function in low body weight patients [16].

TDF is the prodrug of acyclic nucleotide analog tenofovir, which is excreted by both glomerular filtration and active tubular secretion. Tenofovir is considered to cause mitochondrial damage in proximal renal tubular cells [33]. The concentration of tenofovir in the proximal renal tubules could be augmented with the complex interactions of pharmacological, environmental, and genetic factors, including small body weight, consequently resulting in renal tubular dysfunction [34]. Body weight has been identified as an important factor in TDF-related nephrotoxicity not only in clinical trials, but also in *in vitro* and pharmacokinetic studies [35]–[37].

The present study has several limitations. First, because of its retrospective nature, it was not possible to control the baseline characteristics of the enrolled patients. Thus, it is possible that patients with potential risk for TDF-related nephrotoxicity were not prescribed TDF. A proportion of patients treated with ABC had low CD4 count and others were hypertensive, both conditions are known risk factors for renal dysfunction [23], [25]. However, for these reasons, the incidence of TDF-associated renal dysfunction might have been underestimated. Second, the definition of TDF-related nephrotoxicity, especially the criteria used to evaluate proximal renal tubular damage, is not uniformly established in the field and is different in the published studies. Accordingly, we decided to adopt changes in eGFR, instead of parameters for proximal renal tubular damage. Using the eGFR as a marker for TDF-associated renal dysfunction, our results might have underestimated the incidence of TDF-related renal tubular dysfunction. However, the result of this study could be informative to resource-limited settings, where it is difficult to evaluate renal tubular markers. The rational and limitation of adopting more than 25% decrement in eGFR as the criterion for renal dysfunction were discussed in detail in our previous study [16]. Third, our cohort was characterized by the high prevalence of ritonavir-boosted PI use, which is considered by some groups a risk for TDF-related nephrotoxicity [24]. While it is difficult to completely exclude the impact of concurrent ritonavir-boosted PI in this study, it should be noted that the use of ritonavir-boosted PIs did not correlate with renal dysfunction in univariate analysis in this cohort (Table 2). Fourth, the study subjects were mainly men (mostly men who have sex with men and very few injection drug users). Further studies are needed to determine whether the findings of this study are also applicable to females, patients with different route of transmissions, and patients of different racial background.

In conclusion, the present study demonstrated a high incidence of renal dysfunction with TDF use, compared to ABC, among treatment-naïve patients with low body weight. TDF use was identified as an independent risk for renal dysfunction in a statistical model that included TDF as a primary exposure. At 96 weeks, patients with TDF showed greater eGFR decrement than patients treated with ABC. TDF is certainly a drug of choice in the treatment of HIV infection, but the importance of close monitoring of renal function in patients with small body weight, especially those with baseline body weight <60 kg should be emphasized for early detection of TDF-related nephrotoxicity. Further studies are warranted to elucidate the long-term prognosis of renal function with TDF use in patients with low body weight.
